# A Systematic Review of the Clinical Features, Management, and Outcomes in Urethral Squamous Cell Carcinoma

**DOI:** 10.7759/cureus.100444

**Published:** 2025-12-30

**Authors:** Parisa Aijaz, Balqees Ara, Haseeb Faiz, Kulsoom Farooqi Baloch, Rafi Aibani, Abdul Karim Durvesh, Maimoona Khan, Jennifer Collins, Amir S Kamran

**Affiliations:** 1 Internal Medicine, Charleston Area Medical Center, Charleston, USA; 2 Internal Medicine, Mobile Infirmary Medical Center, Mobile, USA; 3 Public Health, Hofstra University, Hempstead, USA; 4 Internal Medicine, Dow University of Health Sciences, Karachi, PAK; 5 Internal Medicine, Dow Medical College, Karachi, PAK; 6 Hematology and Medical Oncology, CAMC Institute of Academic Medicine, Charleston, USA; 7 Hematology and Medical Oncology, Charleston Area Medical Center, Charleston, USA

**Keywords:** genito ureteral tumors, genitourinary oncology, rare, systematic review, urethral squamous cell carcinoma

## Abstract

Primary urethral squamous cell carcinoma (SCC) is a rare and aggressive malignancy, accounting for a minority of genitourinary cancers but presenting significant diagnostic and therapeutic challenges. Due to its rarity, limited data exists to guide standardized management. We aim to systematically review the available literature on urethral SCC, with a focus on epidemiology, clinical presentation, histological characteristics, treatment modalities, and patient outcomes. A comprehensive systematic search of PubMed was conducted for studies published between January 1995 and December 2025, with data extraction performed in accordance with PRISMA guidelines. The inclusion criteria encompassed studies involving patients with histologically confirmed urethral SCC. Retrospective studies and case series/reports were included due to the absence of prospective trials. Primary outcomes included overall survival and disease-specific survival, while secondary outcomes encompassed recurrence rates, treatment-related complications, and quality of life. A total of 27 studies (15 retrospective and 12 case reports/series) were included, representing 2,917 male and 1,292 female patients. The majority of patients were over 60 years of age. The most common presenting symptoms included lower urinary tract obstruction, hematuria, and pelvic mass. High-grade tumors (Grade 3 and 4) were most frequently observed. Treatment modalities varied widely and included transurethral resection, urethrectomy, chemotherapy, and radiotherapy, often in combination. Recurrence was observed in 139 patients, while metastasis was reported in 51. The reported 5- to 10-year survival rates ranged from 30% to 60%. Urethral SCC is a highly aggressive and understudied malignancy with heterogeneous clinical features and treatment practices. The findings underscore the urgent need for standardized staging criteria, prospective multicenter studies, and consensus-driven treatment guidelines. Improved recognition and tailored therapeutic strategies are critical to enhancing outcomes in this rare but impactful cancer.

## Introduction and background

Primary male urethral cancer (PUC) is a rare and aggressive malignancy, accounting for less than 1% of all genitourinary cancers and only 0.02% of all diagnosed cancers [1-3]. Recognized risk factors include a history of urethral strictures, chronic irritation from intermittent self-catheterization, prior urethroplasty, external beam radiation therapy (EBRT), radioactive seed implantation, and chronic urethral inflammation or urethritis due to sexually transmitted infections. Clinically, patients may present with urinary obstruction, irritative voiding symptoms, hematuria, or penile discharge, often accompanied by a palpable mass in the penis or perineum [4-11].

PUC encompasses a variety of histological subtypes. In a Surveillance, Epidemiology, and End Results (SEER) review of 1,615 patients, the most common histologies were transitional cell carcinoma (TCC, 55%), squamous cell carcinoma (SCC, 21.5%), and adenocarcinoma (16.4%). Tumors in the anterior urethra are more prevalent than those in the posterior urethra, with SCC being the predominant subtype in the fossa navicularis and anterior urethra [10-12].

The clinical management and prognosis of primary urethral cancer are closely tied to the tumor’s stage and anatomical location at diagnosis. In the absence of established treatment guidelines, a systematic review is essential to consolidate current evidence, identify gaps in knowledge, and offer evidence-based recommendations for the management of urethral SCC. This review aims to synthesize the literature on urethral SCC, with a focus on its epidemiology, clinical presentation, diagnostic modalities, and therapeutic strategies. By conducting an in-depth analysis of available studies, we aim to refine diagnostic approaches, identify critical prognostic factors, and assess the efficacy of both surgical and non-surgical treatment options.

## Review

Methodology

We conducted a comprehensive systematic review focusing on urethral squamous cell carcinoma (SCC). A detailed search was performed using PubMed, employing combinations of medical terms and keywords, including “urethral squamous cell carcinoma,” “urethral SCC,” “squamous cell carcinoma of the urethra,” and associated treatment modalities such as “management”, “therapy”, “surgery”, “chemotherapy”, “radiotherapy”, and “immunotherapy”.The inclusion criteria encompassed studies involving participants diagnosed with urethral SCC, regardless of age or sex. To ensure comprehensive coverage, no restrictions were applied regarding publication type or language. The search timeline spanned from January 1995 to December 2024, providing a comprehensive overview of the available evidence. Due to the absence of clinical trials dedicated to urethral SCC, the review focused on retrospective studies and case reports. The primary outcomes analyzed included overall survival (OS) and disease-specific survival (DSS). Secondary outcomes included recurrence rates, progression-free survival (PFS), treatment-related complications, and quality of life (QoL). Following the initial search and selection phase, shortlisted studies were reviewed first based on titles, followed by data extraction and subsequent analysis. To maximize our chance of including all relevant studies, we manually checked the reference lists of the included studies. Throughout the review process, a systematic approach was taken, adhering to PRISMA guidelines [13].

**Figure 1:**
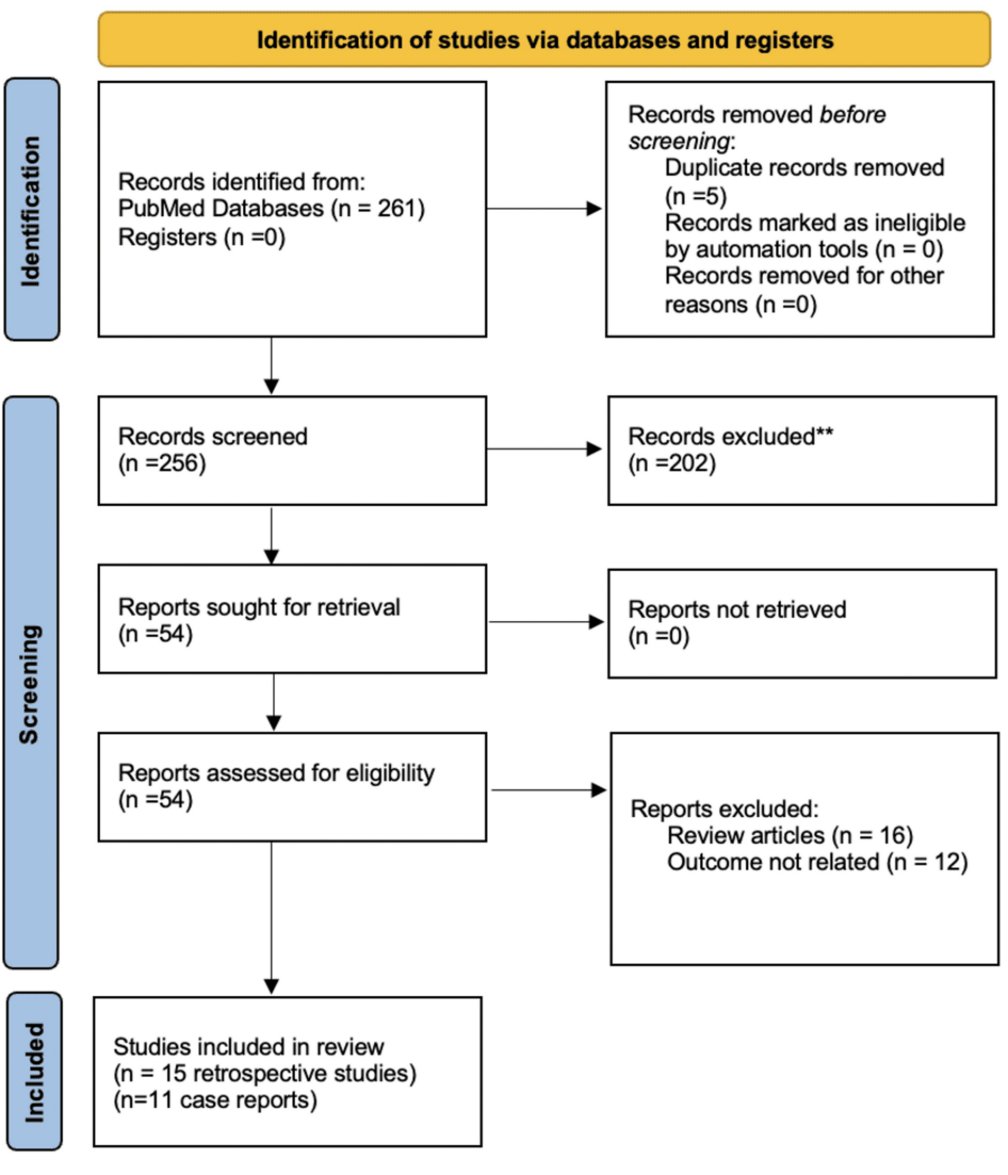
PRISMA flow diagram

**Figure 2:**
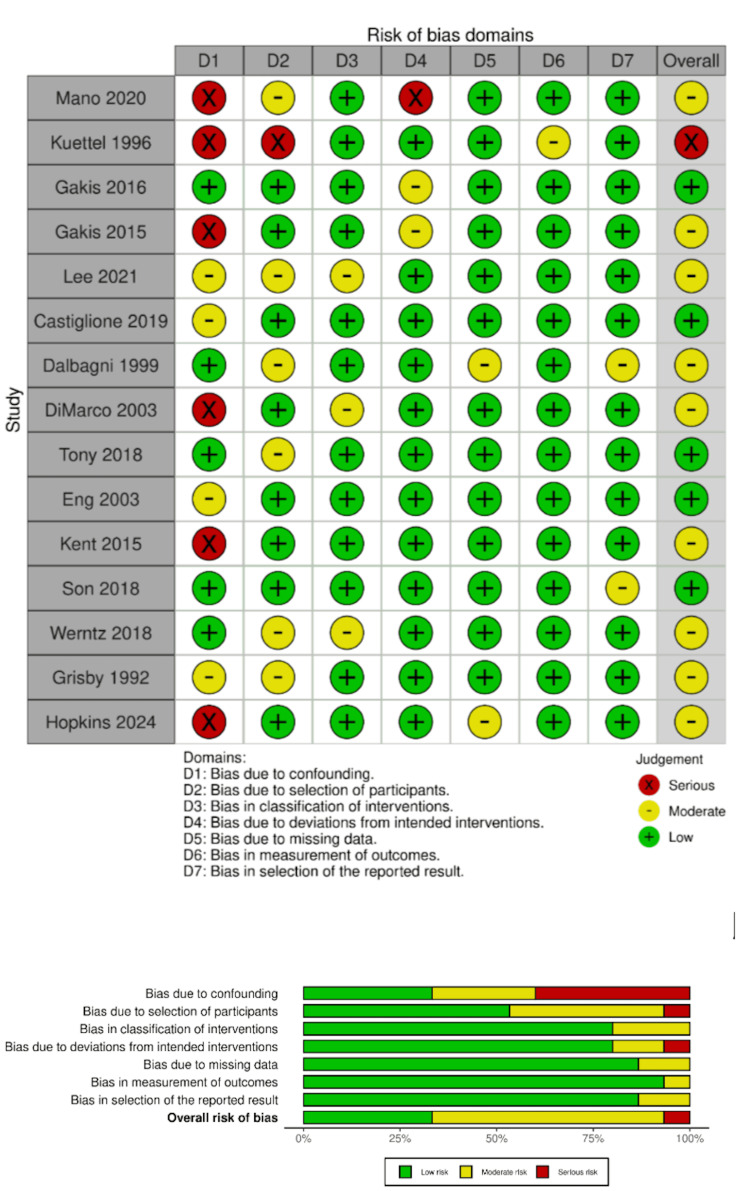
Risk of bias assessment in retrospective studies [16-30]

To assess the risk of bias in retrospective studies, we employed the ROBINS-I tool [14]. Among the 15 included studies, five were rated as “low risk”, nine as “moderate risk”, and one as “high risk” (Table 1, Figure 2). To evaluate the quality of case reports and case series, we utilized the modified Newcastle-Ottawa Scale (NOS) [15]. This tool comprises eight questions that assess four domains: selection, ascertainment, causality, and reporting. We excluded questions four, five, and six, as they primarily pertain to adverse drug reactions and were not applicable to our review. The remaining five questions were used to generate a cumulative score, allowing classification of each study as “low risk,” “medium risk,” or “high risk” of bias. Case reports and case series were considered to have a low risk of bias if they scored four or five, medium risk if they scored three, and high risk if they scored fewer than three. In our analysis, eight studies were rated as “low risk”, and three were rated as “medium risk”. No case reports were identified as high risk (Table 2).

**Table 1:** Risk of bias assessment of retrospective studies using the ROBINS-I tool

Study	D1	D2	D3	D4	D5	D6	D7	Overall bias
Mano et al., 2020 [16]	Serious	Moderate	Low	Serious	Low	Low	Low	Moderate
Kuettel et al., 1996 [17]	Serious	Serious	Low	Low	Low	Moderate	Low	Serious
Gakis et al., 2016 [18]	Low	Low	Low	Moderate	Low	Low	Low	Low
Gakis et al., 2015 [19]	Serious	Low	Low	Moderate	Low	Low	Low	Moderate
Lee et al., 2021 [20]	Moderate	Moderate	Moderate	Low	Low	Low	Low	Moderate
Castiglione et al., 2019 [21]	Moderate	Low	Low	Low	Low	Low	Low	Low
Dalbagni et al., 1999 [22]	Low	Moderate	Low	Low	Moderate	Low	Moderate	Moderate
DiMarco et al., 2003 [23]	Serious	Low	Moderate	Low	Low	Low	Low	Moderate
Tony et al., 2018 [24]	Low	Moderate	Low	Low	Low	Low	Low	Low
Eng et al., 2003 [25]	Moderate	Low	Low	Low	Low	Low	Low	Low
Kent et al., 2015 [26]	Serious	Low	Low	Low	Low	Low	Low	Moderate
Son et al., 2018 [27]	Low	Low	Low	Low	Low	Low	Moderate	Low
Werntz et al., 2018 [28]	Low	Moderate	Moderate	Low	Low	Low	Low	Moderate
Grisby et al., 1992 [29]	Moderate	Moderate	Low	Low	Low	Low	Low	Moderate
Hopkins et al., 2024 [30]	Serious	Low	Low	Low	Moderate	Low	Low	Moderate

**Table 2:** Risk of bias in case reports [31-41]

Study	Does the patient(s) represent(s) the whole experience of the investigator (centre) or is the selection method unclear to the extent that other patients with similar presentation may not have been reported?	Was the exposure adequately ascertained?	Was the outcome adequately ascertained?	Was the follow-up long enough for outcomes to occur?	Are the case(s) described with sufficient details to allow other investigators to replicate the research or to allow practitioners make inferences related to their own practice?	Risk of bias
Lutz et al., 1995 [31]	Yes	Yes	Yes	Yes	Yes	Low risk
Litch et al., 1995 [32]	Yes	Yes	Yes	Yes	Yes	Low risk
Shalev et al., 2002 [33]	Yes	Yes	Yes	No	No	Moderate risk
Paighan et al., 2024 [34]	Yes	Yes	Yes	Yes	Yes	Low risk
Hara et al., 2004 [35]	Yes	Yes	Yes	Yes	Yes	Low risk
Li et al., 2023 [36]	Yes	Yes	Yes	Yes	No	Moderate risk
Coop et al., 2013 [37]	Yes	Yes	Yes	Yes	Yes	Low risk
Baird et al., 2002 [38]	Yes	Yes	Yes	No	Yes	Moderate risk
Bai et al., 2002 [39]	Yes	Yes	Yes	Yes	Yes	Low risk
Tran et al., 1995 [40]	Yes	Yes	Yes	Yes	Yes	Low risk
Johnson et al., 1988 [41]	Yes	Yes	Yes	Yes	Yes	Low risk

Results

The studies included (N = 26) in this review were retrospective studies (N = 15) [16-30], and case reports/case series (N = 11) conducted across North America (N = 18), Europe (N=2), and Asia (N = 5) [31-41]. Both male (N = 2,917) and female (N = 1,292) patients were represented, and the number of patients included varied across studies, as reported in Table 3. In most studies (N = 14), the median age of participants was more than 60 years. 

**Table 3:** Number of patients in each study

Number of patients	Number of studies
0-10	14
11-50	6
>50	6

Presenting Complaints

Patients presented with a variety of symptoms, including obstructive urinary complaints such as urinary frequency, urgency, and hematuria, as well as weight loss and urinary tract infections (Table 4). No significant radiation exposure or history of smoking was reported in the studies included in this systematic review. 

**Table 4:** Presenting complaints

Presenting complaint	N
Obstructive lower urinary tract symptoms	191
Dysuria	8
Palpable pelvic mass/abscess	59
Pelvic pain	14
Spotting	17
Urinary tract infection	20
Irritative symptoms	63
Others	11
Incidental finding	7
Total	390

Types of Cancer

The included studies reported cases of transitional cell/urothelial cell carcinoma (N = 1,716), adenocarcinoma (N = 391), epidermoid carcinoma (N=20), mixed cell carcinoma (N=14), melanoma (N = 4), clear cell carcinoma (N=8), unknown histology (N=14), and other types of carcinoma (N = 190). Tumor grade and stage are described in detail in Table 5. Reported tumor sizes ranged from 21-40 mm (N = 47) to >40 mm (N = 6). 

**Table 5:** Tumor grading and staging

Parameter	N
Grade	
1	173
2	515
3	1,212
4	489
X	538
Total	2,927
Stage of Tumor	
M0	157
M1	8
N0	2,282
N1	273
N2	249
Nx	877
T2	737
T3	581
T4	388
Tis	24
Tx	1,088
Total	666

Treatment

Depending on the grade and staging of the tumor, patients underwent various treatments, including but not limited to transurethral resection, urethrectomy, chemotherapy, and radiation therapy (Table 6). Some patients received adjuvant/neoadjuvant chemotherapy as well, and the regimens included 5-fluorouracil (5-FU) (N = 4), cisplatin with gemcitabine/5-FU (N = 14), 5-FU combined with Mitomycin-C (N = 40), and others (N = 6). 

**Table 6:** Treatment modalities

Treatment	Number
Transurethral resection	397
Partial urethrectomy	33
Penectomy with/without cystectomy	83
Radical prostatectomy with/without panurethrectomy	37
Cystectomy	2
Radiotherapy	52
Chemotherapy	30
Combination therapy	69
Others	21
Total	724

Outcomes

The mean follow-up period reported by the studies ranged from 1-10 years (N = 11), with two studies reporting follow-up periods of more than 10 years (N = 2). Survival status at the end of follow-up was reported in only a few studies, with 32 patients alive and 34 deceased. The reported causes of death included disease metastasis, cerebrovascular event, and other causes. Some studies also noted disease recurrence and metastasis (Table 7). 

**Table 7:** Outcomes

Disease recurrence and metastasis	Number of patients
Recurrence	139
Metastasis	51

**Figure 3:**
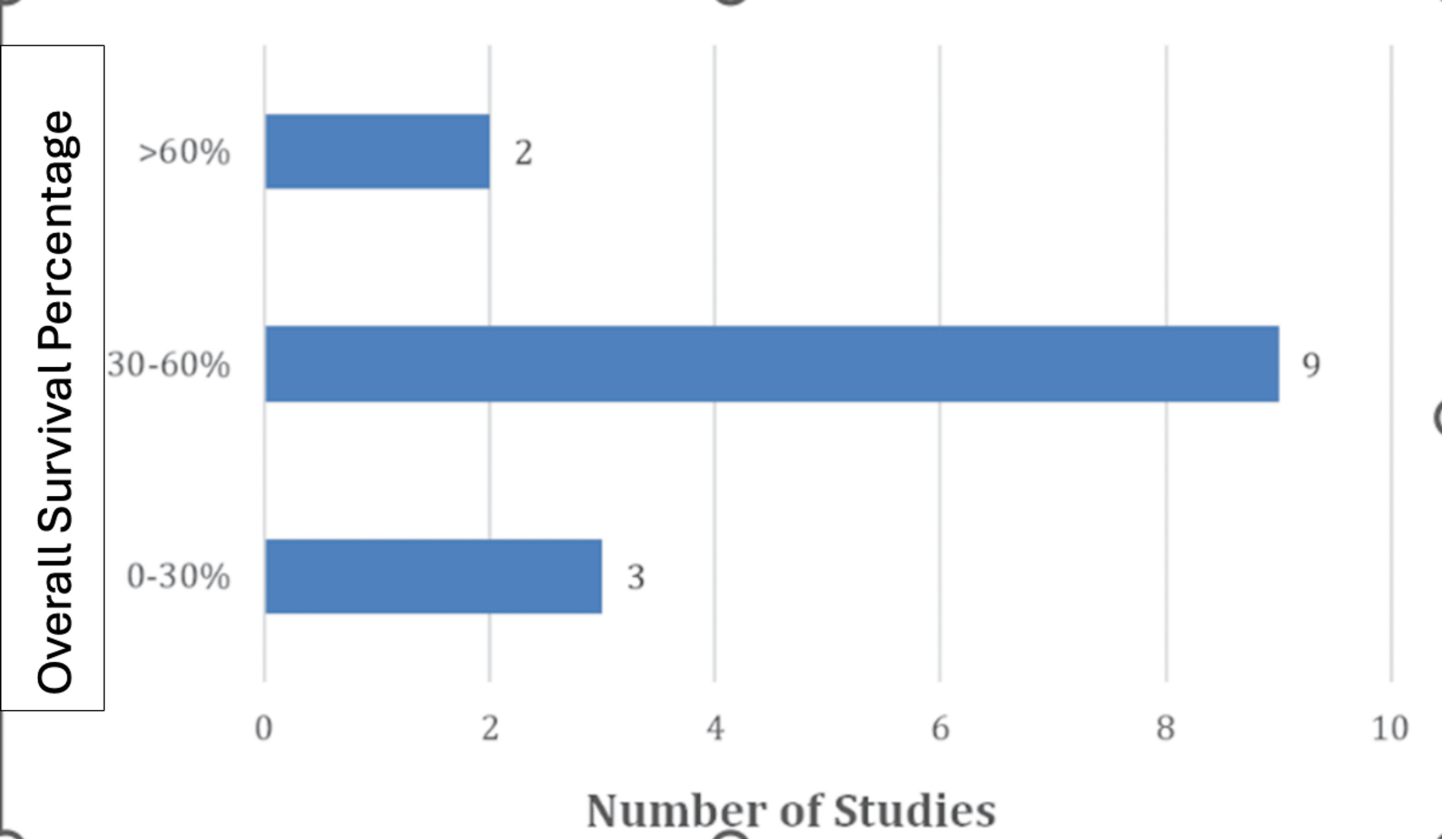
Overall survival percentage

Overall survival percentages varied by study; however, most studies reported 5- to 10-year survival rates between 30% and 60% (Figure 3).

Discussion 

Urethral SCC is an uncommon but clinically important cancer known for its aggressive nature and the diagnostic and therapeutic challenges it presents. Despite representing less than 1% of all genitourinary cancers, urethral SCC accounts for approximately 60%-80% of primary urethral malignancies, underscoring its clinical significance [42]. Effective management of urethral SCC requires a multidisciplinary strategy, prioritizing precise staging, complete surgical removal with negative margins, and careful handling of the complex anatomical structures involved. 

This review examined 26 studies on urethral squamous cell carcinoma (SCC), including case reports and case series (n = 11) as well as retrospective analyses (n = 15). The studies originated predominantly from North America (18 studies), followed by Asia (5 studies), and Europe (2 studies), highlighting geographic differences and suggesting a scarcity of comprehensive global data. Notably, there was a significant disparity in gender representation, with a substantially larger cohort of 2,917 male patients compared to 1,292 females. The observed difference could be due to a higher true incidence of urethral SCC in males or inconsistencies in reporting, underscoring the need for further research to explore gender-related epidemiological patterns [42,43]. In 14 studies, the median age of participants was more than 60 years. This predominance of older age groups suggests that urethral SCC might be influenced by factors associated with aging, such as long-term exposure to risk factors or natural physiological changes over time [1, 2]. In terms of patient numbers, most studies were relatively small, with nearly half (n = 12) involving 10 or fewer patients. Medium-sized studies (11-50 patients) were less frequent (n = 6), and only a minority of studies (n = 6) examined cohorts larger than 50 patients. This pattern underscores a significant limitation, namely the lack of extensive, well-designed studies capable of providing clearer insights into clinical features, prognostic indicators, and treatment effectiveness [44,45]. 

Our analysis of the presenting complaints revealed a broad spectrum of clinical presentations in urethral SCC, emphasizing its diagnostic complexity and variability. Patients diagnosed with urethral SCC most commonly presented with obstructive lower urinary tract symptoms (LUTS), including urinary frequency, urgency, and hematuria, reported in 191 patients. Irritative symptoms were also frequently observed, affecting 63 patients. Additionally, a palpable pelvic mass or abscess (59 patients), urinary tract infections (20 patients), and spotting (17 patients) were notable presenting complaints. Less common presentations included pelvic pain (14 patients), dysuria (8 patients), incidental findings (7 patients), and other unspecified symptoms (11 patients). Interestingly, none of the studies included in this review reported notable associations with radiation exposure or smoking history, both of which are common risk factors for other urothelial cancers. This absence suggests that urethral SCC may have a different etiological profile, warranting further research into alternative risk factors and underlying disease mechanisms [43]. 

Among the various histological types of urethral carcinoma, transitional cell carcinoma (TCC) or urothelial carcinoma was the most frequently reported type, occurring in 1,716 cases. Adenocarcinoma was the second most common, with 391 cases. Less common histological types included epidermoid carcinoma reported in 20 cases, mixed cell carcinoma in 14 cases, clear cell carcinoma in 8 cases, and melanoma in 4 cases. Other carcinoma types accounted for 190 cases, while tumor histology remained unspecified or unknown in 14 cases. The frequent occurrence of transitional cell carcinoma aligns with trends seen in other urothelial malignancies. However, the significant presence of adenocarcinoma and rare histological types underscores the heterogeneity of urethral carcinoma, necessitating tailored diagnostic and treatment approaches. The analysis of tumor grades revealed that most cases presented with high-grade tumors, such as Grade 3 in 1,212 patients and Grade 4 in 489 patients. Lower grades were less common, with Grade 2 observed in 515 patients and Grade 1 identified in only 173 patients. Tumor grade remained unspecified in 538 cases. In terms of tumor size, most tumors measured between 21 and 40 mm (47 cases), while larger tumors exceeding 40 mm were relatively rare, reported in only 6 cases. This variation in tumor size may suggest delays in diagnosis or differences in tumor aggressiveness. Overall, these findings indicate that urethral carcinoma is typically diagnosed at advanced stages and is associated with high-grade pathology. The significant proportion of high-grade tumors may be attributed to either the aggressive biological nature of urethral carcinoma or delays in diagnosis, underscoring the need for heightened clinical awareness and earlier detection efforts [43,44]. Additionally, variations in tumor staging and grading reporting highlight the necessity of standardized pathology guidelines to ensure more accurate prognostic assessments and improved patient care [1,42]. Future studies should aim to clarify these discrepancies and identify key factors driving the development and progression of different histological subtypes. 

The treatment of urethral carcinoma exhibited considerable variation, emphasizing the need for a tailored approach based on disease severity [28]. Transurethral resection was the most commonly performed procedure, conducted in 397 patients, primarily for initial management or symptom control. In contrast, more extensive surgical interventions, such as penectomy (with or without cystectomy) in 83 patients and radical prostatectomy (with or without panurethrectomy) in 37 patients, were less frequently employed but highlight the necessity of aggressive surgical strategies in advanced cases [39,43]. Radiotherapy was administered to 52 individuals, while chemotherapy was utilized in 30 individuals, reflecting the acknowledged role of multimodal treatments in managing locally advanced or aggressive tumors. Additionally, combination therapy, reported in 69 patients, underscores the importance of an integrated treatment approach designed to improve survival outcomes and control disease progression [39,42]. The combination of mitomycin-C and 5-FC was the most frequently utilized chemotherapy regimen, given to 40 patients, indicating either its widespread clinical adoption and/or proven therapeutic efficacy [42]. The wide range of therapeutic approaches seen highlights the urgent need for tailored treatment plans based on specific tumor grade, stage, and patient features, as well as the present absence of generally recognized recommendations. These results highlight how urgent it is to use prospective, multicenter research to provide standardized, evidence-based treatment strategies. Such initiatives are required to improve treatment results, create clear recommendations for the care of urethral cancer, and increase the clarity of clinical decision-making [1]. 

These outcomes underscore the aggressive nature of urethral SCC and highlight several critical issues in the management of urethral SCC. The significant recurrence rate (139 cases) suggests that, despite treatment, many patients experience disease relapse, which could be attributed to incomplete tumor resection, the aggressive nature of SCC, or limitations in current therapeutic options. The relatively lower number of metastasis cases (51) may indicate that while the disease is highly recurrent locally, systemic spread occurs less frequently, potentially providing a window for more effective localized interventions. The broad range of survival rates (30%-60% over 5-10 years) further underscores the variability in patient outcomes, likely influenced by factors such as tumor stage at diagnosis, treatment modalities used, and individual patient characteristics. This variability suggests that while some patients respond well to treatment, others may have limited benefit, pointing to gaps in optimal disease management. Moreover, the lack of long-term follow-up in many studies limits a comprehensive understanding of disease progression, survival predictors, and late treatment effects. These results emphasize the pressing need for more standardized, multicenter research to refine treatment protocols, improve early detection strategies, and establish more effective follow-up guidelines. Additionally, efforts should focus on identifying prognostic markers that can help stratify patients based on risk, allowing for more personalized therapeutic approaches. Such disparities further emphasize the critical need for larger, standardized clinical studies and clear prognostic criteria to improve management practices and enhance patient survival outcomes [2,12]. 

Limitations of this review include the retrospective nature of most included studies, small sample sizes, and the predominance of case reports, potentially introducing selection bias and limiting the generalizability of findings. Future research on urethral SCC needs to overcome a number of shortcomings highlighted in the present literature. Limited knowledge of etiological variables, noteworthy diversity in treatment regimens, inconsistency in tumor staging and grading, gender and regional inequities, and a preponderance of small-scale research are some of these limitations. Additionally, there were a few reports of typical urothelial cancer risk factors, including smoking and radiation exposure, suggesting that urethral SCC may have different underlying processes. Therefore, even when a patient does not have a conventional risk profile, clinicians need to keep a high index of suspicion for urethral SCC. Larger, prospective, multi-center studies employing standardized techniques to improve data comparability and generalizability will be necessary to overcome these constraints. Furthermore, new environmental, genetic, or other risk factors unique to urethral SCC should be the focus of future studies. Accurately assessing long-term results also requires longer and more consistent follow-up times. In the end, developing evidence-based clinical standards that are the result of thorough, multicenter research endeavors would greatly enhance patient survival outcomes, treatment decision-making, and diagnostic accuracy. 

## Conclusions

Urethral SCC remains a rare, yet formidable malignancy characterized by aggressive behavior, diagnostic complexity, and a lack of standardized management pathways. Despite advances in oncologic care, the scarcity of high-quality, large-scale studies continues to hinder the development of clear clinical guidelines. This systematic review highlights the heterogeneity of clinical presentations, histological subtypes, and treatment approaches, emphasizing the urgent need for multidisciplinary collaboration and evidence-based strategies. Future multicenter research is essential to define standardized treatment protocols, improve early detection, and ultimately enhance patient outcomes in this understudied disease.
